# Knowledge, Indications and Willingness to Take Pre-Exposure Prophylaxis among Transwomen in San Francisco, 2013

**DOI:** 10.1371/journal.pone.0128971

**Published:** 2015-06-03

**Authors:** Erin C. Wilson, Harry Jin, Albert Liu, H. Fisher Raymond

**Affiliations:** 1 Center for Public Health Research, San Francisco Department of Public Health, San Francisco, California, United States of America; 2 Bridge HIV, San Francisco Department of Public Health, San Francisco, California, United States of America; David Geffen School of Medicine at UCLA, UNITED STATES

## Abstract

Safe and effective HIV prevention strategies are needed for transwomen. Transwomen in the US have a 34 times greater odds of being infected with HIV than all adults age 15-49, and in San Francisco, California 42.4% of transwomen are estimated to be infected with HIV. Pre-exposure prophylaxis (PrEP) is the first biomedical intervention with promise for reducing HIV acquisition in transwomen. However, little is known about whether transwomen know about PrEP, are taking PrEP and would be good candidates for PrEP based on their risk profile and behaviors. A population-based dataset was analyzed to determine how many transwomen in San Francisco knew about PrEP by the end of 2013 – more than a year after iPrex results demonstrated efficacy of PrEP in preventing HIV. We found that of 233 transwomen, only 13.7% had heard of PrEP. Transwomen who were living with HIV compared to those who were HIV-negative, and those who recently injected drugs compared to non-injection drug users were more likely to have heard of PrEP. Based on CDC guidelines for PrEP among MSM and IDU, 45 (30.2%) transwomen of the 149 HIV-negative transwomen in the sample were candidates for PrEP. This estimate based on CDC criteria is arguably low. Given that almost half of transwomen in San Francisco are living with HIV, this findings points to a need for further consideration of PrEP criteria that are specific and tailored to the risks for HIV faced by transwomen that are different from MSM and injection drug users. Research to scale up access and test the effectiveness of PrEP for transwomen is also urgently needed.

## Introduction

Transgender women (transwomen) are part of a persistent HIV epidemic in the United States (US) for which few interventions have offered hope of delivering effective HIV prevention approaches [1]. Research for over a decade has demonstrated elevated HIV infection rates among transwomen; over a quarter in the US are HIV-positive [2–4]. A recent systematic review summarized the odds of HIV infection among transwomen at 48.8 greater odds compared to the general population of reproductive age adults in all countries with data [5]. This figure is high even compared to other key populations at risk for HIV such as men who have sex with men in the Americas with a 33.3-fold odds of HIV infection (18.7-fold in Asia, 3.8-fold in Africa, and 1.3-fold in Eastern Europe) and 13.5-fold for female sex workers (FSW) [6]. Data on the odds of HIV infection for transwomen are lacking, in part, because transgender people are not identified in National HIV surveillance data.

Pre-exposure prophylaxis (PrEP) is the first biomedical intervention with promise for reducing HIV acquisition in transwomen. The iPrEx randomized controlled trial in MSM and TGW demonstrated a 44% reduction in HIV incidence among individuals who received once daily emtricitabine/tenofovir (FTC/TDF), and an estimated >90% efficacy among those with detectable blood drug levels.[7,8] Based on compelling data from iPrEx and other PrEP trials,[9,10] the US Food and Drug Administration approved FTC/TDF for the prevention of sexually acquired HIV infection in July 2012 [11,12]. In May 2014, the CDC issued comprehensive guidelines for the use of PrEP as a powerful tool to reduce new infections among men and women at substantial risk of HIV acquisition [13–16]. To date, few studies have identified whether transwomen are knowledgeable of PrEP and are willing to take medication as a way to prevent acquisition of HIV [17].

TEACH2 (Transwomen empowered to Advance Community Health, second round surveillance) was an HIV behavioral risk survey conducted with 234 transwomen in San Francisco from August-December 2013 using Respondent Driven Sampling (RDS) [18–20]. In this article, we identify the level of awareness of PrEP among transwomen, and examine whether demographic factors are predictive of PrEP awareness. In preparation for a PrEP implementation program in San Francisco, we use the recent comprehensive CDC guidelines on PrEP [16] to measure how many transwomen would be eligible for this program and describe baseline demographics, risk characteristics and knowledge of PrEP in this population.

## Materials and Methods

### Study Sample and Recruitment

The study was conducted between August and December 2013. Following RDS standards, recruitment started with 12 ethnically/racially diverse seeds, or highly socially networked individuals who were at least 18 years of age and who identified as transwomen [19]. Seeds were chosen based on fitting the diversity criteria and they were motivated, enthusiastic, and willing to try to recruit their peers, and who we hoped were in high regard among peers, thus motivating recruits to continue recruiting peers (i.e., “socio-metric stars”) [21]. Seeds recruited others from their immediate social networks, which through long-chain referral eventually crossed-over and reach diverse networks. Starting with diverse seeds facilitated the successful recruitment of an RDS sample [22]. Seeds were recruited from local CBOs and through referrals from the original TEACH study. Most initial seeds (8/12) were recruited from the first round surveillance study (i.e. TEACH [23]) seed population and from referrals. Seeds and enrolled participants received coupons (three to five each) to recruit through word of mouth other transwomen into the study from their respective social networks[23]. The coupon return rate was 33.1%. This study reached sample stability in key variables and satisfied other ideal RDS criteria [11, 12]. Each study participant was screened for study eligibility before enrollment. Individuals were eligible for the study if they (1) self-identified as male-to-female or transfemale, (2) were aged 18 years or older, and (3) reported living in San Francisco. Verbal informed consent was obtained to protect participant privacy and minimize risks for loss of confidentiality by not having a signed informed consent as the only record of participation. Verbal consent was noted on the tablet computer before starting the behavioral survey. Waiver of signed consent and all study procedures received approval from the Committee on Human Research at the University of California San Francisco.

Participants received $50 for participation in the survey and HIV testing. Each participant received $10 for each successful recruit up to 5 people each. Instant HIV testing was offered to all participants regardless of self-reported HIV status (INSTI HIV-1 Antibody Test, bioLytical Laboratories). Positive rapid HIV tests were confirmed using a secondary rapid finger prick test (The Clearview HIV 1/2 STAT-PAK, Alere). All participants who tested positive were referred to the San Francisco Department of Public Health Linkage Integration Navigation Comprehensive Services (LINCS) program which provides and coordinates comprehensive HIV care for newly tested positives and known positives who are currently out of care. Data were collected with a standardized, interviewer-administered questionnaire via handheld-computer tablets. An in depth description of the study sampling and recruitment is described in Wilson et al. 2014 [24].

### Measures and Analysis

The study collected information on socio-demographics, including gender identity, race, whether the participant was born in the U.S. or not, monthly income, level of education, and housing status. For this analysis, we used univariate statistics (number, percentage) to describe the basic demographic and risk characteristics of individuals with knowledge of PrEP and conducted chi-squared analyses to determine differences between those with and without prior reported knowledge of PrEP. PrEP knowledge was assessed with a yes/no/don’t know response to the following question: “Researchers are studying whether antiretroviral medicines could possibly be taken to prevent HIV infection. Before today, have you ever heard of people who do not have HIV taking antiretroviral medicines, to keep from getting HIV?” For those who had heard of PrEP, a follow-up question was asked- “Would you be willing to take anti-HIV medicines every day to lower your chances of getting HIV?” For the sexual risk behaviors and partnership characterstics, participants were asked to report on up to 5 sexual partners from the past six months, including each partner’s age, gender identity, race/ethnicity, what type of partner each person was (i.e. primary, casual or commercial), where the participant met each partner, sexual behavior, HIV status and whether that person was an injection drug user. Injection drug use was assessed with the question,” Have you ever injected any drug that was not prescribed to you by your doctor or health care provider?.”

We then analyzed the number of transwomen in San Francisco who would be eligible to receive PrEP using the CDC recommended PrEP indications for MSM and IDU [16] (no recommendations were provided by the CDC for transwomen). Based on the CDC guidelines for MSM, transwomen were considered PrEP eligible if they met the following criteria = (1) adult, (2) HIV-negative, (3) had any male partners in last 6 months, (4) were not in primary partnership with a HIV-negative man and had one of the following: any anal sex w/o condoms in last 6 months OR any STI diagnosed in last 6 months OR was in a primary partnership with someone who is HIV-positive. For the latter criteria, transwomen were considered being in a primary partnership with someone who is HIV-positive only if they reported one primary partnership and that primary partner was HIV-positive. Based on the CDC guidelines for IDU, transwomen were considered PrEP eligible if they met the following criteria = (1) adult, (2) HIV-negative, (3) any non-prescription IDU in last 6 months, excluding hormones/fillers and one of the following: any sharing of drug using equipment in past 6 months, OR is at sexual risk (i.e. fit criteria for HIV-negative transwoman sexual risk). We did not have data for the CDC criteria based on prior methadone, buprenorphine, or suboxone treatment program in past 6 months.

## Results

Among the 250 transwomen who were screened for eligibility, 93.6% were deemed eligible and agreed to participate, leaving a study sample N of 233. Overall, 32 transwomen, or just 13.7% had heard of PrEP, and only one person of those 32 who had heard of PrEP was willing to use PrEP for HIV prevention. There were few significant demographic and no risk behavior differences between those with and without PrEP knowledge (Table 1). The only exceptions were for HIV status and injections drug use (IDU). Significantly more transwomen living with HIV than those not living with HIV knew about PrEP (20% vs. 10.5%, p = 0.05). There were also significantly more transwomen with a history of injection drug use who knew about PrEP compared to those with no history of injection drug use (45.5% vs. 8.5%, p<0.01). There was tendency towards higher awareness among those with a HIV+ primary partner (p = 0.05).

**Table 1:** Correlates of PrEP knowledge among transwomen, San Francisco.

	Overall	Knowledge of PrEP (n = 32)	NO knowledge of PrEP	Chisq	p-value
Race				5.0592	0.28
Latina	74	14(18.9)	60 (81.1)		
White	58	4 (6.9)	54 (93.1)		
Black	81	10 (12.4)	71 (83.6)		
Asian	7	1 (14.3)	6 (85.7)		
Other	13	3 (23.1)	10 (76.)		
Age					
18–20	5	1 (20.0)	4 (80.0)		
21–29	22	1 (4.6)	21 (95.4)	2.8905	0.58
30–39	38	7 (18.4)	31 (81.6)		
40–49	84	13 (15.5)	71 (84.5)		
50+	84	10 (11.9)	74 (88.1)		
Gender					
Female	102	9 (8.8)	93 (91.2)	4.0905	0.13
Transfemale	123	21 (17.1)	102 (82.9)		
Other +	8	2 (25.0)	6 (75.0)		
Sexual Orientation					
Heterosexual	114	16 (14.0)	98 (86.0)	1.9022	0.59
Homosexual	63	9 (14.3)	54 (85.7)		
Bisexual	45	7 (15.6)	38 (84.4)		
Other	11	0 (0.0)	11 (100.0)		
Monthly Income					
0–417	31			1.9367	0.75
418–833	48	4 (12.9)	27 (87.1)		
834–1250	108	4 (8.3)	44 (91.7)		
1251–1667	12	16 (14.8)	92 (85.2)		
1668+	33	2 (16.7)	10 (83.3)		
Housing					
Own/rent	120	19 (15.8)	101 (84.2)	1.8513	0.76
Live with partner/family/friend	9	1 (11.1)	8 (88.9)		
SRO	60	8 (13.3)	52 (86.7)		
Homeless	34	3 (8.8)	31 (91.2)		
Other	10	1 (10.0)	9 (90.0)		
Currently Using hormones					
Yes	159	21 (13.2)	138 (86.8)	0.1171	0.73
No	74	11 (14.9)	63 (85.1)		
Self reported HIV status					
Negative	153	16 (10.5)	137 (89.5)	4.0375	0.05*
Positive	80	16 (20.0)	64 (80.0)		
HIV Test result					
Negative	149	16 (10.7)	133 (89.3)	3.1305	0.08
Positive	84	16 (19.1)	68 (20.9)		
Number of male sex partners in last 6 months					
6 or less	181	23 (12.7)	158 (87.3)	0.8166	0.37
More than 6	51	9 (17.7)	42 (92.3)		
Partner Type, past 6 months					
Primary only	46	11 (23.9)	35 (76.1)	8.6258	0.03*
Casual and/or exchange	85	14 (16.5)	71 (83.5)		
Primary and casual/exchange	43	3 (7.0)	40 (93.0)		
No partners in past 6	59	4 (6.8)	55 (93.2)		
Condomless anal sex in last 6 months					
Yes	147	18 (12.2)	129 (87.8)	0.7453	0.39
No	86	14 (16.3)	72 (83.7)		
Condomless sex with partner who is IDU					
Yes	19	3 (15.8)	16 (84.2)	0.0738	0.79
No	214	29 (13.6)	185 (86.4)		
Any STI in past 6 months					
Yes	28	3 (10.7)	25 (89.3)	0.2449	0.62
No	205	29 (14.2)	176 (85.8)		
Primary partner who is HIV-positive					
Yes	17	5 (29.4)	12 (70.6)	3.8044	0.05
No	216	27 (12.5)	189 (87.5)		
Injection drug use in last 12 months					
Yes	33	15 (45.5)	18 (54.5)	32.6505	<0.01**
No	200	17 (8.5)	183 (91.5)		
Has shared injecting equipment in last 6 months					
Yes	5	2(40.0)	3 (60.0)	2.9754	0.08
No	228	30 (13.2)	198 (86.8)		

*P value<0.05

** P value <0.01

+ Additional genders given were ambigender, questioning, genderqueer, “additional sex and gender”

A total of 36.1% of participants or 84 people tested HIV positive, leaving 149 potential HIV-negative transwomen to be assessed for recommended PrEP use. We found that 45 (30.2%) transwomen from the TEACH2 study who were HIV-negative, including 8 who were also IDU, would be eligible to receive PrEP in San Francisco based on recent CDC guidelines.

Housing was the only significant factor differentiating groups transwomen who would and would not be candidates for PrEP (Table 2). Those who lived in an SRO or were homeless were much more likely to be candidates for PrEP compared to those who owned or rented their places (25% of those who lived in SROs and 23.5% of those who were homeless were candidate for PrEP, while only 15% of those who owned or rented their housing were candidates; p = 0.02). Similar to the overall study, Latinas and African Americans (n = 58 and n = 66, respectively) and those who identified as transgender rather than female (n = 27 and n = 17, respectively) made up the largest proportions within racial/ethnic and gender identities groups of those who would meet CDC recommendations for PrEP among HIV-negatives. Of those who were taking hormones, 18.2% would meet CDC PrEP eligibility criteria. Of those who had heard of PrEP, 15.6% would be candidates for it. No participants who would be candidates for PrEP said they were willing to take it.

**Table 2:** Demographics of transwomen who would be good candidates for use of PrEP, San Francisco, 2013.

	Transwomen who are *not* candidates for PrEP (n = 187)	HIV-negative transwomen PrEP candidates(n = 45)	Chisq	P value
Age (Mean, SD)	45.9, 10.7	40.6, 10.7		
Race				
Latina	58 (78.4)	16 (21.6)	0.6773	0.95
White	48 (82.8)	10 (17.2)		
African American/Black	66 (81.5)	15 (18.5)		
Asian	6 (85.7)	1 (6.3)		
Other	10 (76.9)	3 (83.1)		
Gender				
Female	85 (83.3)	17 (16.7)	1.2461	0.54
Transfemale	96 (78.0)	27 (22.0)		
Other	7 (87.5)	1 (12.5)		
Income				
0–417	23 (74.2)	8 (25.8)	4.1672	0.38
418–833	37 (77.1)	11 (22.9)		
834–1250	92 85.2)	16 (14.8)		
1251–1667	8 (66.6)	4 (33.4)		
1668+	27 (81.8)	6 (78.2)		
Housing				
Own/rent	91 (85.0)	16 (15.0)	12.2111	0.02
Live with partner/family/friend	18 (81.8)	4 (18.2)		
SRO	45 (75.0)	15 (25.0)		
Homeless	26 (76.5)	8 (23.5)		
Other	8 (80.0)	2 (20.0)		
Currently taking hormones				
No	58 (78.4)	16 (21.6)	0.3708	0.54
Yes	130 (81.8)	29 (18.2)		
Heard of Prep				
No	161 (80.1)	40 (19.9)	0.3238	0.57
Yes	27 (84.4)	5 (15.6)		
Would take Prep				
No	187 (80.6)	45 (19.4)	0.2404	0.62
Yes	1 (1.0)	0 (0.0)		
Seen for mental health				
No	90 (75.6)	26 (84.4)	1.4250	0.23
Yes	98 (83.8)	19 (16.2)		
Non-injected substance use				
No	109 (82.0)	24 (18.0)	0.3198	0.57
Yes	79 (79.0)	21 (21.0)		

• HIV-negative transwomen based on MSM guidelines from CDC = (1) adult, (2) HIV-negative, (3) any male partners in last 6 months, (4) not in primary partnership with HIV-negative man and one of the following: (a) any anal sex w/o condoms in last 6 months OR (b) any STI diagnosed in last 6 months OR (c) is in a primary partnership with someone who is HIV-positive

• HIV-negative transwoman IDU (1) adult, (2) HIV-negative, (3) any non-prescription IDU in last 6 months, excluding hormones/fillers and one of the following: (a) any sharing of drug using equipment in past 6 months, OR (b) is at sexual risk (i.e. fit criteria for HIV-negative transwoman sexual risk)

## Discussion

Only 30% of transwomen in this sample would be good candidates for PrEP based on the CDC criteria for MSM and injection drug users, which is arguably a low estimate and does not represent actual need. Due to the absence of incidence data for HIV among transwomen, it is unclear what the transmission dynamics of HIV are for this population. However, much like that found among African American/Black MSM, risk for HIV among transwomen may be more closely tied to risky sexual networks and may be more independent of individual risk behaviors [25,26]. This finding points to a need for further consideration of PrEP criteria that are specific and tailored to the risks for HIV faced by transwomen that are different from MSM and injection drug users. One important consideration in this effort is how to safely and effectively open the criteria so that as many transwomen at risk for HIV acquisition can access PrEP so as to curb the extreme epidemic faced by this population, particularly in places like San Francisco.

These data also provide a strong case the need for more education about PrEP in this population. The vast majority of transwomen in this study were unaware of PrEP, which is consistent with studies of MSM immediately before and post iPrEx results [27–29]. However, the low level of knowledge among transwomen in this study is markedly lower than even the lowest knowledge among MSM even before the iPrEx results. [30] This low level of knowledge is especially surprising in San Francisco where local clinical trials have been conducted and iPrEx results were widely publicized in local press. [7] Though these data do not point to racial/ethnic or other disparities in PrEP knowledge, as has been a prior concern [28,31], findings do point to an overall disparity in knowledge among transwomen compared to MSM.

Transwomen have not been a large component of many biomedical intervention trials, which may be one reason transwomen are less knowledgeable about PrEP. And since there are very few social services or clinics in San Francisco for transpeople, there are few venues where transwomen may encounter educational materials and marketing around PrEP. Finally, transwomen have a number of competing priorities in their lives that may make protection from HIV a low priority [32]. For those who are at risk and interested in protection, there may be unique health concerns that are not currently addressed in the roll out of PrEP as it related to transwomen. Golub et al. (2013) specifically called for studies that investigate the unique concerns and health issues facing transwomen that are different from those for MSM when rolling out biomedical HIV prevention interventions. For transwomen, concerns about reactions or decreased efficacy of hormone therapy may be of particular concern. The majority of transwomen in this study were currently using hormones at the time they were surveyed. More broadly, many transwomen have significant barriers to access and utilization of health care[33,34]. Some of those barriers to engagement in care include misinformation about treatment and prevention modalities,[35] substance use and mental health issues,[36,37] trauma/violence,[37,38] lack of social support,[36] housing instability,[39] distrust of medical institutions and lack of access to trans-friendly providers,[34,40] and HIV stigma and transphobia. [41–43] These more general barriers to care may certainly impact transwomen’s interest in obtaining PrEP, which is currently only available in a traditional health care paradigm.

Findings also show that almost one-fifth of transwomen in San Francisco would be good candidates for PrEP given their risk profile and HIV negative status. Based on population size estimates for transwomen in San Francisco, this means that 290 transwomen would be candidates for PrEP [44]. Universal coverage of PrEP for eligible transwomen in San Francisco may be entirely feasible based on these estimates. Increased marketing, especially at the three city-based transgender-specific health clinics and numerous other clinics with expertise in transgender health, could significantly impact knowledge and uptake of PrEP. Transwomen are confronted by a wide array of barriers to engagement in care, including misinformation about treatment and prevention modalities,[35] concerns about side effects and possible interaction of medications with hormones,[45] substance use and mental health issues,[36,37] trauma/violence,[37,38] lack of social support,[36] housing instability,[39] distrust of medical institutions and lack of access to trans-friendly providers,[34,40] and HIV stigma and transphobia.[41–43] Due to the many competing priorities transwomen face, interventions to increase PrEP uptake and adherence must address a number of these barriers. Studies that address the potential interactions between PrEP and hormones may further interest and uptake of this prevention tool. Making PrEP accessible in transgender-specific health care clinics with trusted providers may help address concerns of transwomen interested in taking PrEP. Demonstration projects have been recommended to address a number of these implementation issues, and given the high risk and importance of HIV for transwomen, such studies would be highly warranted for this population. [46,47]

This study in not without limitations. This study was not intended to exclusively investigate the knowledge and use of PrEP among transwomen and only asked such questions to determine if there was a relationship with HIV risk. This study is also cross sectional in nature and we therefore cannot determine temporal trends in behavior and health outcomes. Finally, due to questionnaire constraints, an explanation of PrEP and its safety and efficacy demonstrated in PrEP trials was not provided to participants, which may also explain the low level of PrEP interest in this survey.

## Conclusions

Despite challenges with this study, we have provided the first population-based study of transwomen in San Francisco that measures PrEP knowledge in this population and provides data to inform efforts to reach out to transwomen to promote use of this important prevention tool. An important next step is to conduct interventions the increase awareness and uptake of PrEP among transwomen and address potentials barriers to adherence. Much like heterosexual couples in various places in Africa and MSM in the US and elsewhere, transwomen are at high risk for HIV and stand to benefit from this important HIV prevention tool.
